# Scope and Limits of an Anamnestic Questionnaire in a Control-Induced Low-Endemicity Helminthiasis Setting in South-Central Côte d’Ivoire

**DOI:** 10.1371/journal.pone.0064380

**Published:** 2013-06-03

**Authors:** Thomas Fürst, Mamadou Ouattara, Kigbafori D. Silué, Dje N. N’Goran, Lukas G. Adiossan, Isaac I. Bogoch, Yao N’Guessan, Siaka Koné, Jürg Utzinger, Eliézer K. N’Goran

**Affiliations:** 1 Department of Epidemiology and Public Health, Swiss Tropical and Public Health Institute, Basel, Switzerland; 2 University of Basel, Basel, Switzerland; 3 Unité de Formation et de Recherche Biosciences, Université Félix Houphouët-Boigny, Abidjan, Côte d’Ivoire; 4 Centre Suisse de Recherches Scientifiques en Côte d’Ivoire, Abidjan, Côte d’Ivoire; 5 Programme National de Lutte contre la Schistosomiase, les Géohelminthiases et la Filariose Lymphatique, Abidjan, Côte d’Ivoire; 6 Hôpital Général de Taabo, Taabo Cité, Côte d’Ivoire; 7 Divisions of Internal Medicine and Infectious Diseases, Toronto General Hospital, Toronto, Canada; The George Washington University Medical Center, United States of America

## Abstract

**Background:**

Schistosomiasis and soil-transmitted helminthiasis are two high-burden neglected tropical diseases. In highly endemic areas, control efforts emphasize preventive chemotherapy. However, as morbidity, infection, and transmission begin to decrease, more targeted treatment is likely to become more cost-effective, provided that comparatively cheap diagnostic methods with reasonable accuracy are available.

**Methodology:**

Adults were administered an anamnestic questionnaire in mid-2010 during a cross-sectional epidemiological survey in the Taabo health demographic surveillance system in south-central Côte d’Ivoire. Questions pertaining to risk factors and signs and symptoms for schistosomiasis and soil-transmitted helminthiasis were included. The individuals’ helminth infection status and their belonging to three different anthelmintic treatment groups were compared with the questionnaire results (i) to inform the local health authorities about the epidemiological and clinical footprint of locally prevailing helminthiases, and (ii) to explore the scope and limits of an anamnestic questionnaire as monitoring tool, which eventually could help guiding the control of neglected tropical diseases in control-induced low-endemicity settings.

**Principal Findings:**

Our study sample consisted of 195 adults (101 males, 94 females). We found prevalences of hookworm, *Trichuris trichiura*, *Schistosoma haematobium*, and *Schistosoma mansoni* of 39.0%, 2.7%, 2.1%, and 2.1%, respectively. No *Ascaris lumbricoides* infection was found. Helminth infection intensities were generally very low. Seven, 74 and 79 participants belonged to three different treatment groups. Multivariable logistic regression models revealed statistically significant (p<0.05) associations between some risk factors, signs, and symptoms, and the different helminth infections and treatment groups. However, the risk factors, signs, and symptoms showed weak diagnostic properties.

**Conclusions/Significance:**

The generally low prevalence and intensity of helminth infection in this part of south-central Côte d’Ivoire indicates that recent control efforts have turned our study area into a low endemicity setting. Our anamnestic questionnaire had low sensitivity and specificity to identify infected individuals or treatment groups.

## Introduction

Schistosomiasis and soil-transmitted helminthiasis are two high-burden neglected tropical diseases [1]. Burden estimates, as expressed in disability-adjusted life years (DALYs), range from 1.7 to 70 million DALYs for schistosomiasis [1]–[8], and from 2.9 to 39 million DALYs for soil-transmitted helminthiasis [1], [3], [4], [8], [9]. The awareness for these high disease burdens have increased in the past few years, but despite the fact that control efforts are going to scale, a variety of high and low endemicity zones remain around the globe [10], [11].

The main strategy to combat the diseases in highly endemic areas is morbidity control. Once morbidity has decreased, the control strategies foresee a progressive shift toward infection and transmission control, surveillance and case detection and, ultimately, local elimination [12]–[14]. Consequently, the diagnosis, treatment, and control strategies have to be adapted [13], [15], [16]. For instance, in high endemicity areas, preventive chemotherapy (i.e., regular treatment of high-risk groups without prior diagnosis [17]) using available, safe, and efficacious drugs that are inexpensive or donated by pharmaceutical companies, is the most widely used strategy [17], [18]. However, as morbidity, infection, and transmission begin to decrease, more targeted treatment might become more cost-effective [19], provided that comparatively cheap diagnostic methods with reasonable accuracy are available [13]. Such diagnostics have to consider the changes in parasite ecology that increasingly occur due to the scale-up of preventive chemotherapy and the expansion of more integrated control strategies, which tackle multiple helminth species simultaneously [18], [20] and also on non-drug-based routes (e.g., providing clean water and improved sanitation) [21], [22]. Hence, control-induced low-endemicity settings have become the new reality of helminth epidemiology in many areas.

Simple, rapid, inexpensive, and culturally adapted questionnaires have been considered as useful diagnostic tools to screen communities and guiding control interventions over the past years [23]. Regarding schistosomiasis, for example, school-based questionnaires proved useful for identification of high-risk communities of *Schistosoma haematobium* [24]. Indeed, school prevalence of self-reported blood in urine correlates well with the prevalence of *S. haematobium* [24]. A simple anamnestic questionnaire, including questions on signs, symptoms, and water contact patterns, allowed individual diagnosis of *S. japonicum* [25]. However, adaptation for *S. mansoni* and other helminth infections proved to be more difficult [13], [24], [26] and the usefulness of anamnestic questionnaires seems to be restrained in regions with naturally low helminth endemicity as noticeable signs and symptoms are rare. Further research is therefore needed to determine the scope and limits of anamnestic questionnaires when they are employed over the course of control programs, which are characterized by declining morbidity and prevalence rates [13], [23], [27].

In this paper, we report our experience from a cross-sectional survey carried out in mid-2010 as part of a prospective longitudinal monitoring of people’s malaria and neglected tropical diseases status in the Taabo health demographic surveillance system (HDSS) in south-central Côte d’Ivoire. The study area represents an epidemiological situation, which is influenced by helminth control activities [28]. The two objectives of the survey were (i) to assess risk factors, signs, and symptoms related to schistosomiasis and soil-transmitted helminthiasis in order to inform the local health authorities about the epidemiological and clinical footprint of these two helminthiasis, and (ii) to explore the scope and limits of an anamnestic questionnaire as monitoring tool, which eventually could help guiding the control of neglected tropical diseases in control-induced low-endemicity settings.

## Methods

### Ethics Statement

The study protocol was cleared by the institutional research commissions of the Centre Suisse de Recherches Scientifiques en Côte d’Ivoire (CSRS; Abidjan, Côte d’Ivoire) and the Swiss Tropical and Public Health Institute (Swiss TPH; Basel, Switzerland). Ethical clearance was obtained from the Comité National d’Ethique et de la Recherche (CNER) in Côte d’Ivoire (reference no. 1086 MSHP/CNER) and the ethics committee in Basel (EKBB; reference no. 316/08).

The study was integrated in the second annual parasitological survey and preventive chemotherapy campaign in the Taabo HDSS in June 2010. District and village authorities and the general public were informed about the purpose, procedures, potential risks and benefits of the annual survey, treatment, and the current questionnaire study. Written informed consent was obtained from all participants of the present study. Everybody living in the area of the Taabo HDSS was invited for a free anthelmintic treatment with ivermectin (∼200 µg/kg using a dose pole) and albendazole (400 mg single oral dose), irrespective of the infection status or participation in the present study [29], [30]. Praziquantel (40 mg/kg using a dose pole), was administered half a year later for individuals aged 5 years and above in the course of a preventive chemotherapy campaign against schistosomiasis [29]. Medical staff accompanied the survey, anthelmintic treatment, and follow-up.

### Study Area and Population

The study area and population have been described elsewhere [31]. In brief, the Taabo HDSS was established in 2008 around Lake Taabo in south-central Côte d’Ivoire. It covers most of the rural sub-district of Taabo with a surface area of approximately 1,000 km^2^. Since 2012, the Taabo HDSS is a member center of the INDEPTH Network (see http://www.indepth-network.org). The main station of the Taabo HDSS is located in Taabo Cité, the only small urban settlement in the Taabo HDSS, 160 km north-west of Abidjan. Most people in the region cultivate yams, manioc, and banana mainly for subsistence. Coffee and cacao are farmed as cash crops. Furthermore, there is a minority of fishermen, artisans, shopkeepers, and businessmen.

Lake Taabo is a man-made impoundment resulting from damming up the Bandama River in the late 1970s for hydroelectric power generation [32]. Hence, the study area underwent major ecologic and demographic transformation, which favored the spread of schistosomiasis [32], [33] and might have influenced patterns of other helminth infections [34], [35] and malaria [36]. Before the establishment of the Taabo HDSS, different studies provided sporadic anthelmintic treatment to some village communities. Therefore, the development of more systematic and integrated disease control measures, particularly annual preventive chemotherapy campaigns against helminthiases and a strengthening of the health system became specific objectives of the Taabo HDSS.

### Data Collection

The parasitological data for the present study were obtained in the frame of the second cross-sectional survey and preventive chemotherapy campaign (carried out once every year) in the Taabo HDSS. While everybody living in the area of the Taabo HDSS was invited to participate in the preventive chemotherapy campaign, the members of approximately 7% of all registered households were selected for the epidemiological survey based on a stratified random sampling procedure. They were asked to provide fresh morning stool and urine samples. The samples were transferred to the laboratory of the general hospital in Taabo Cité and analyzed the same day by experienced laboratory technicians using standardized, quality-controlled techniques [37], [38]. In short, duplicate Kato-Katz thick smears were prepared with 41.7 mg of stool and microscopically examined for *S. mansoni* and soil-transmitted helminths (*Ascaris lumbricoides*, *Trichuris trichiura*, and hookworm). In order to obtain infection intensities as expressed in eggs per gram of stool (EPG), the sum of the helminth-specific egg counts from the two Kato-Katz thick smears were multiplied by a factor 12 [29]. Urine samples were vigorously shaken, 10 ml subjected to a filtration, and the filters, after adding a drop of iodine Lugol, microscopically examined for *S. haematobium*. Five percent of the Kato-Katz thick smears and the urine filters were re-examined by a senior technician. In case of disagreement, the slides were read a third time and the results discussed among the technicians until agreement was reached.

All heads of households and, if possible, a second adult household member of the opposite sex were eligible for the present study. On the day of the epidemiological survey, all eligible individuals were invited to complete a questionnaire on risk factors, signs, and symptoms pertaining to different neglected tropical diseases (Text S1) with the assistance of a trained field enumerator or supervisor of the Taabo HDSS. Questions were either asked in French or translated and explained in any of the local languages (Baoulé, Dioula, or Senoufo). Our questionnaire was carefully developed from previously employed questionnaires in Côte d’Ivoire [39]–[42], further adapted during discussions with health personnel and Taabo HDSS staff and pre-tested in a nearby village.

Additional sociodemographic data on the individual and household level, including information on sex, age, education, main occupation, relationship with the respective head of household, type of housing, and availability of facilities were readily available from the Taabo HDSS database. For further details on the field and laboratory procedures, the reader is referred to Fürst et al. (2012) [31]. All data from the present study can be obtained from the authors upon request.

### Statistical Analysis

Data were double-entered and cross-checked in EpiInfo version 3.5.1 (Centers for Disease Control and Prevention; Atlanta, United States of America), and analyzed in STATA version 10.1 (STATA Corp.; College Station, United States of America). Participants for the present study were purposefully sampled, as described in the previous section, with no formal sample size calculation. Only individuals with complete datasets were included in our final analysis.

Age was stratified into three groups, namely (i) 18–40 years, (ii) 41–60 years, and (iii) >60 years. Educational levels were classified as (i) none, (ii) primary, and (iii) secondary or higher. Occupation was grouped into farmer, fisherman and hunter, housewife, builder and artisan, and being employed in the tertiary sector (includes driver, housekeeper, watchman, merchant, trader, hairdresser, gastronome, healer, nurse, teacher, student, office worker, and policeman). The parasitological results were classified according to infection intensities as expressed in EPG (for *S. mansoni* and soil-transmitted helminths) and eggs/10 ml of urine (for *S. haematobium*), according to World Health Organization (WHO) guidelines [29]. Furthermore, treatment groups were established, relying on the parasitological results and current WHO recommendations [18], i.e., *S. haematobium* and *S. mansoni* infections in the praziquantel treatment group (Tx1), *A. lumbricoides*, *T. trichiura*, and hookworm infections in the benzimidazole treatment group (Tx2), and all single or multiple infections with *S. haematobium*, *S. mansoni*, *A. lumbricoides*, *T. trichiura*, and hookworm in the overarching praziquantel and benzimidazole treatment group (Tx3).

Initially, χ^2^ test statistics and Fisher’s exact test, as appropriate, were used to identify univariable associations between helminth infections and treatment groups, respectively, and reported risk factors, signs, and symptoms. Risk factors, signs, and symptoms significantly associated (p<0.05) were then included as explanatories in a multivariable logistic regression, again with the outcomes helminth infections and treatment groups. A stepwise backward elimination procedure was performed, removing the explanatory variable with the highest p-value one after the other, as long as the Akaike information criterion (AIC) was decreasing. Associations between anamnestic questions and treatment groups were considered as the correct treatment may be more important than exact species identification for control program managers.

Either each of the remaining and significantly associated (p<0.05) explanatories on their own or all of them combined were used as diagnostic variables to predict helminth infections and treatment group specific classifications of individuals. In case of combining diagnostic variables, a scoring approach was adopted. All significantly associated risk factors, signs, and symptoms were coded 0 or 1 with the higher score indicating elevated odds for being infected with the respective helminth or belonging to a certain treatment group. The scores from all significantly associated risk factors, signs, and symptoms were then summed up to obtain each participant’s combined score. Sensitivity (i.e., proportion of true-positives recognized as positives), specificity (i.e., proportion of true-negatives recognized as negatives), positive predictive value (PPV; i.e., probability that a positively tested individual is a true-positive), and negative predictive value (NPV; i.e., probability that a negatively tested individual is a true-negative) were used to assess the diagnostic performance of each significantly associated risk factor, sign, and symptom on its own and of the combined score at different cut-off levels.

## Results

### Study Cohort and Compliance

**Figure 1:**
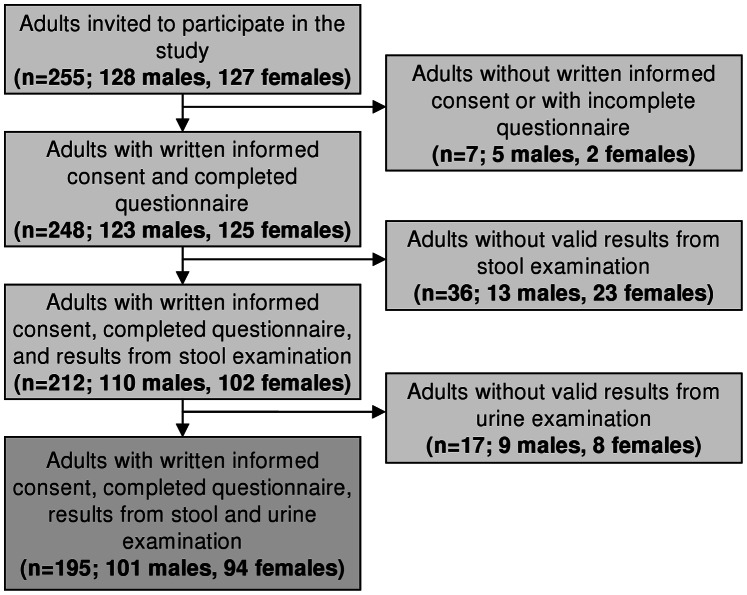
Study participation and compliance. The study was carried out in June 2010 in the Taabo health demographic surveillance system, south-central Côte d’Ivoire.

Details of our study cohort have been described elsewhere [31]. Overall, 255 adults were invited (128 males and 127 females; Figure 1). Sixty individuals were excluded (27 males and 33 females); seven had no written informed consent or failed to have complete questionnaire results, whereas 53 had no valid results from the parasitological examination, mainly because they lacked sufficiently large stool and/or urine samples for diagnostic work-up. Our final study sample consisted of 195 adults (101 males and 94 females) with details of the sociodemographic characteristics summarized in Table 1.

**Table 1:** Sociodemographic characteristics among the 195 study participants with complete questionnaire and parasitological data.

Sociodemographic characteristics		Number of people (%)
Sex	Male	101 (51.8)
	Female	94 (48.2)
Age (years)	18–40	107 (54.9)
	41–60	66 (33.9)
	>60	22 (11.3)
Education	None	106 (54.4)
	Primary	41 (21.0)
	Secondary or higher	48 (24.6)
Main occupation	Farmer	123 (63.1)
	Tertiary sector^a^	49 (25.1)
	Housewife	13 (6.7)
	Builder and artisan	8 (4.1)
	Fisherman and hunter	2 (1.0)

The study was carried out in June 2010 in the Taabo health demographic surveillance system, south-central Côte d’Ivoire.

a Including driver, housekeeper, watchman, merchant, trader, hairdresser, gastronome, healer, nurse, teacher, student, office worker, and policeman.

### Parasitological Results

Table 2 shows the parasitological results. We found very low prevalence for *S. haematobium* and *S. mansoni* (2.1% for each schistosome species). The prevalence for hookworm and *T. trichiura* were 38.5% and 2.6%, whereas no *A. lumbricoides* were found. Most helminth infections were of low intensity. Seven, 76 and 81 participants belonged to Tx1, Tx2, and Tx3, respectively.

**Table 2:** Prevalence and intensity of helminth infections among the 195 study participants.

Parasite	Infection intensity^a^	Number of people (%)
*Schistosoma haematobium*	Negative	191 (97.9)
	Light	4 (2.1)
	Heavy	0
*Schistosoma mansoni*	Negative	191 (97.9)
	Light	3 (1.6)
	Moderate	1 (0.5)
	Heavy	0
Tx1 (*Sh*+*Sm*)	Negative	188 (96.4)
	Positive	7 (3.6)
Hookworm	Negative	120 (61.5)
	Light	72 (36.9)
	Moderate	3 (1.6)
	Heavy	0
*Trichuris trichiura*	Negative	190 (97.4)
	Light	4 (2.1)
	Moderate	1 (0.5)
	Heavy	0
*Ascaris lumbricoides*	Negative	195 (100.0)
	Light	0
	Moderate	0
	Heavy	0
Tx2 (Hw+*Tt*+*Al*)	Negative	119 (61.0)
	Positive	76 (39.0)
Tx3 (*Sh*+*Sm*+Hw+*Tt*+*Al*)	Negative	114 (58.5)
	Positive	81 (41.5)

The study was carried out in June 2010 in the Taabo health demographic surveillance system, south-central Côte d’Ivoire.

*Al*, *A. lumbricoides*; Hw, hookworm; *Sh*, *S. haematobium*; *Sm*, *S. mansoni*; *Tt*, *T. trichiura*; Tx1, treatment group 1 (i.e., praziquantel against schistosomiasis); Tx2, treatment group 2 (i.e., benzimidazole against soil-transmitted helminthiasis); Tx3, treatment group 3 (i.e., praziquantel and benzimidazole against schistosomiasis and soil-transmitted helminthiasis, respectively).

a Infection intensities according to WHO guidelines [29].

### Results from Univariable Analysis

Based on univariable χ^2^ test statistics and Fisher’s exact test, we identified one risk factor and one symptom that were significantly associated with *S. haematobium* infection, three risk factors that were significantly associated with *S. mansoni*, 18 risk factors that were significantly associated with hookworm infection, and two risk factors that were significantly associated with *T. trichiura* infections (Table 3). Four risk factors and two symptoms were significantly associated with Tx1, 18 risk factors with Tx2, and 11 risk factors with Tx3, as shown in Table 4.

**Table 3:** Risk factors, signs, and symptoms significantly (p<0.05) associated with helminth infections, as determined by univariable analysis.

Parasite	Risk factor, sign, and symptom	p-value^a^
*Schistosoma haematobium*	Vertigo	0.026
	Worm infections considered frequent in household	0.027
*Schistosoma mansoni*	Occupation: farmer	0.018
	Occupation: housewife	0.023
	Drinking water: rain	0.011
Hookworm	Occupation: farmer	0.022
	Occupation: tertiary sector	0.027
	Tile or carpet flooring	0.011
	Type of toilet: WC	0.048
	Open defecation	0.014
	Natural water contact: washing oneself	0.010
	Natural water contact: cooking	0.014
	Natural water contact: washing children	0.033
	Natural water contact: cultivating rice	0.019
	Natural water contact: religious worship	0.045
	Drinking water: natural water body	0.001
	Drinking water: rain	0.042
	Drinking water: fountain	0.011
	Using soap for washing clothes	0.015
	Using soap for washing dishes	0.024
	Washing hands after defecation	0.003
	Washing hands when returning from work	0.004
	Worm infections considered frequent in household	0.003
*Trichuris trichiura*	Using soap for washing oneself	0.018
	Having a cat	0.048

The study was carried out in June 2010 in the Taabo health demographic surveillance system, south-central Côte d’Ivoire.

a Univariable analysis, using χ^2^ test statistics and Fisher’s exact test, as appropriate.

**Table 4:** Risk factors, signs, and symptoms significantly (p<0.05) associated with treatment groups, as determined by univariable analysis.

Treatment group^a^	Risk factor, sign, and symptom	p-value^b^
Tx1	Occupation: farmer	0.011
	Occupation: builder and artisan	0.028
	Drinking water: rain	0.005
	Headache	0.049
	Vertigo	0.018
	Worm infections considered frequent in household	0.020
Tx2	Occupation: farmer	0.015
	Occupation: tertiary sector	0.018
	Tile or carpet flooring	0.010
	Type of toilet: WC	0.047
	Open defecation	0.022
	Natural water contact: washing oneself	0.010
	Natural water contact: cooking	0.022
	Natural water contact: washing children	0.035
	Natural water contact: cultivating rice	0.010
	Drinking water: natural water body	0.001
	Drinking water: rain	0.042
	Drinking water: fountain	0.017
	Using soap for washing clothes	0.015
	Using soap for washing dishes	0.025
	Washing hands after defecation	0.005
	Washing hands when returning from work	0.004
	Having poultry	0.043
	Worm infections considered frequent in household	0.004
Tx3	Occupation: tertiary sector	0.044
	Tile or carpet flooring	0.009
	Uncemented latrine	0.028
	Natural water contact: washing oneself	0.011
	Natural water contact: cultivating rice	0.011
	Drinking water: natural water body	0.003
	Drinking water: fountain	0.018
	Using soap for washing clothes	0.018
	Washing hands after defecation	0.005
	Washing hands when returning from work	0.002
	Worm infections considered frequent in household	0.002

The study was carried out in June 2010 in the Taabo health demographic surveillance system, south-central Côte d’Ivoire.

a Tx1, treatment group 1 (i.e., praziquantel against schistosomiasis); Tx2, treatment group 2 (i.e., benzimidazole against soil-transmitted helminthiasis); Tx3, treatment group 3 (i.e., praziquantel and benzimidazole against schistosomiasis and soil-transmitted helminthiasis, respectively).

b Univariable analysis, using χ^2^ test statistics and Fisher’s exact test, as appropriate.

### Results from Multivariable Analysis

Many of the univariably significantly associated risk factors, signs, and symptoms presented in Tables 3 and 4 dropped out when subjecting them to multivariable logistic regression with a stepwise backward elimination procedure, as shown in Table 5. The only remaining significant explanatory variable for *S. mansoni* infection was being a housewife (odds ratio (OR) = 10.3, 95% confidence interval (CI) 1.1–102.1), whereas all explanatories for *S. haematobium* were eliminated. People belonging to the praziquantel treatment group (Tx1) had an increased risk for vertigo (OR = 16.9, 95% CI 1.2–34.6).

**Table 5:** Risk factors, signs, and symptoms significantly (p<0.05) associated with parasites and treatment groups, as determined by multivariable logistic regression modeling.

Parasite ortreatment group^a^	Risk factor, sign, and symptom	Adjusted oddsratio [95% CI]^b^	Sensitivity (%)[95% CI]^b^	Specificity (%)[95% CI]^b^	Positivepredictive value [95% CI]^b^	Negative predictivevalue [95% CI]^b^
*S. mansoni*	Housewife	10.3 [1.1, 102.1]	50.0 [43.0, 57.0]	94.2 [91.0, 97.5]	15.4 [10.3, 20.5]	98.9 [97.4, 100.0]
Hookworm	Natural water contact: cultivating rice	2.6 [1.2, 5.7]	29.3 [22.9, 35.7]	85.0 [80.0, 90.0]	55.0 [48.0, 62.0]	65.8 [59.2, 72.5]
	Drinking water: fountain	0.4 [0.2, 0.8]	29.3 [22.9, 35.7]	51.7 [44.6, 58.7]	27.5 [21.2, 33.8]	53.9 [46.9, 60.9]
	Using soap for washing clothes	0.1 [0.02, 0.6]	89.3 [85.0, 93.7]	1.7 [0.1, 3.5]	36.2 [29.5, 43.0]	20.0 [14.4, 25.6]
	Worm infections considered frequent in household	4.6 [1.4, 14.6]	18.7 [13.2, 24.1]	95.0 [91.9, 98.1]	70.0 [63.6, 76.4]	65.1 [58.5, 71.8]
*T. trichiura*	Using soap for washing oneself	0.05 [0.01, 0.4]	60.0 [53.1, 66.9]	3.7 [1.0, 6.3]	1.6 [0.1, 3.4]	77.8 [71.90, 83.6]
	Having a cat	10.6 [1.3, 85.8]	40.0 [33.1, 46.9]	93.2 [89.6, 96.7]	13.3 [8.6, 18.1]	98.3 [96.5, 100.0]
Tx1	Vertigo	16.9 [1.2, 34.6]	85.7 [80.8, 90.6]	61.2 [54.3, 68.0]	7.6 [3.9, 11.3]	99.1 [97.8, 100.0]
Tx2	Natural water contact: cultivating rice	2.9 [1.3, 6.4]	30.3 [23.8, 36.7]	85.7 [80.8, 90.6]	57.5 [50.6, 64.4]	65.8 [59.2, 72.5]
	Drinking water: fountain	0.4 [0.2, 0.9]	30.3 [23.8, 36.7]	52.1 [45.1, 59.1]	28.8 [22.4, 35.1]	53.9 [46.9 60.1]
	Using soap for washing clothes	0.1 [0.02, 0.6]	89.5 [85.2, 93.8]	1.7 [0.1, 3.5]	36.8 [30.0, 43.5]	20.0 [14.4, 25.6]
	Washing hands when returning from work	0.5 [0.2, 0.9]	25.0 [18.9, 31.1]	53.8 [46.8, 60.8]	25.7 [19.5, 31.8]	52.9 [45.9, 59.9]
	Worm infections considered frequent in household	4.4 [1.4, 14.1]	18.4 [13.0, 23.9]	95.0 [91.9, 98.0]	70.0 [63.6, 76.4]	64.6 [57.9, 71.3]
Tx3	Natural water contact: cultivating rice	2.8 [1.3, 6.2]	29.6 [23.2, 36.0]	86.0 [81.1, 90.8]	60.0 [53.1, 66.9]	63.2 [56.5, 70.0]
	Drinking water: fountain	0.4 [0.2, 0.8]	30.9 [24.4, 37.4]	51.8 [44.7, 58.8]	31.3 [24.7, 37.8]	51.3 [44.3, 58.3]
	Using soap for washing clothes	0.1 [0.02, 0.8]	90.1 [85.9, 94.3]	1.8 [0.01, 3.6]	39.5 [32.6, 46.3]	20.0 [14.4, 25.6]
	Washing hands when returning from work	0.4 [0.2, 0.8]	24.7 [18.6, 30.7]	52.6 [45.6, 59.6]	27.0 [20.8, 33.3]	49.6 [42.6, 56.6]
	Worm infections considered frequent in household	5.4 [1.7, 17.9]	18.5 [13.1, 24.0]	95.6 [92.7, 98.5]	75.0 [68.9, 81.1]	62.3 [55.5, 69.1]

The study was carried out in June 2010 in the Taabo health demographic surveillance system, south-central Côte d’Ivoire. Stepwise backward elimination was performed, removing explanatory variables with the highest p-value one at the time, as long as the Akaike information criterion (AIC) decreased. Diagnostic indicators for each risk factor, sign, and symptom are indicated.

a Tx1, treatment group 1 (i.e., praziquantel against schistosomiasis); Tx2, treatment group 2 (i.e., benzimidazole against soil-transmitted helminthiasis); Tx3, treatment group 3 (i.e., praziquantel and benzimidazole against schistosomiasis and soil-transmitted helminthiasis, respectively).

b CI, confidence interval.

People cultivating rice (OR = 2.6, 95% CI 1.2–5.7) and considering worm infections as something that occurs frequently in their household (OR = 4.6, 95% CI 1.4–14.6) were at a higher risk for hookworm infection. On the other hand, people who use fountains as a primary source of drinking water (OR = 0.4, 95% CI 0.2–0.8) and those who use soap for washing clothes (OR = 0.1, 95% CI 0.02–0.6) had low odds of hookworm infection. Regarding *T. trichiura*, having a cat as domestic animal was associated with a high odds of infection (OR = 10.6, 95% CI 1.3–85.8), whereas using soap for washing oneself showed a low odds of infection (OR = 0.05, 95% CI 0.01–0.4). People cultivating rice (OR = 2.9, 95% CI 1.3–6.4) and considering worm infections as something that occurs frequently in their household (OR = 4.4, 95% CI 1.4–14.1) showed higher odds and people reporting fountains as an important source of drinking water (OR = 0.4, 95% CI 0.2–0.9), using soap for washing clothes (OR = 0.1, 95% CI 0.02–0.6), and washing their hands when returning from work (OR = 0.5, 95% CI 0.2–0.9) had lower odds to belonging to the benzimidazole treatment group (Tx2).

Cultivating rice (OR = 2.8, 95% CI 1.3–6.2) or considering worm infections as frequent in the household (OR = 5.4, 95% CI 1.7–17.9) were associated with high odds, whereas fountains as an important source of drinking water (OR = 0.4, 95% CI 0.2–0.8), using soap for washing clothes (OR = 0.1, 95% CI 0.02–0.8), or washing hands when returning from work (OR = 0.4, 95% CI 0.2–0.8) were associated with low odds of schistosomiasis and soil-transmitted helminthiasis, and therefore belonging to the treatment group Tx3.

### Diagnostic Properties of Risk Factors, Signs, and Symptoms

If each of the significant risk factors, signs, and symptoms revealed from the multivariable analysis was considered as diagnostic variable on its own, estimated sensitivity and specificity showed that at least one of the two diagnostic indicators was ≤50% with the exception of vertigo and belonging to Tx1 (sensitivity = 85.7%, 95% CI 80.8–90.6%; specificity = 61.2%, 95% CI 54.3–68.0%) (Table 5). However, when calculating the respective specificity for vertigo as a diagnostic indicator for Tx1, a high number of false-positives was masked by the high number of correctly identified negatives in the studied low schistosomiasis prevalence sample, as revealed by the inferior corresponding PPV of 7.6% (95% CI 3.9–11.3%).

When considering the combined score, the only combinations that achieved values >50% for sensitivity, specificity, and the predictive values occurred for Tx2 and Tx3 at the cut-off level >1 (Table 6). By increasing the cut-off levels for predicting positive cases, the number of false-positives decreased and the number of false-negatives increased, consequentially leading to lower sensitivities and higher specificities.

**Table 6:** Diagnostic properties of a combined score at different cut-off levels in the diagnosis of helminth infections and treatment groups.

Parasite ortreatment group^a^	Combined score cut-offlevels for predictingpositive cases	Number of predictedpositive cases	Sensitivity (%)[95% CI]^b^	Specificity (%)[95% CI]^b^	Positive predictivevalue[95% CI]^b^	Negative predictivevalue [95% CI]^b^
*S. mansoni*	>0	13	50.0 [43.0, 57.0]	94.2 [91.0, 97.5]	15.4 [10.3, 20.5]	98.9 [97.4, 100.0]
Hookworm	>0	142	86.7 [81.9, 91.4]	35.8 [29.1, 42.6]	45.8 [38.8, 52.8]	81.1 [75.6, 86.6]
	>1	36	33.3 [26.7, 40.0]	90.8 [86.8, 94.9]	69.4 [63.0, 75.9]	68.6 [62.0, 75.1]
	>2	7	9.3 [5.3, 13.4]	100.0 [100.0, 100.0]	100.0 [100.0, 100.0]	63.8 [57.1, 70.6]
	>3	0	n.a.	n.a.	n.a.	n.a.
*T. trichiura*	>0	23	60.0 [53.1, 66.9]	89.5 [85.2, 93.8]	13.0 [8.3, 17.8]	98.8 [97.3, 100.0]
	>1	1	20.0 [14.4, 25.6]	100.0 [100.0, 100.0]	100.0 [100.0, 100.0]	97.9 [95.9, 99.9]
Tx1	>0	79	85.7 [80.8, 90.6]	61.2 [54.3, 68.0]	7.6 [3.9, 11.3]	99.1 [97.8, 100.0]
Tx2	>0	168	97.4 [95.1, 99.6]	21.0 [15.3, 26.7]	44.1 [37.1, 51.0]	92.6 [88.9, 96.3]
	>1	108	75.0 [68.9, 81.1]	57.1 [50.2, 64.1]	52.8 [45.8, 59.8]	78.2 [72.4, 84.0]
	>2	25	25.0 [18.9, 31.1]	95.0 [91.9, 98.0]	76.0 [70.0, 82.0]	66.5 [59.8, 73.1]
	>3	5	6.6 [3.1, 10.1]	100.0 [100.0, 100.0]	100.0 [100.0, 100.0]	62.6 [55.8, 69.4]
	>4	0	n.a.	n.a.	n.a.	n.a.
Tx3	>0	168	96.3 [93.7, 99.0]	21.1 [15.3, 26.8]	46.4 [39.4, 53.4]	88.9 [84.5, 93.3]
	>1	108	75.3 [69.3, 81.4]	58.8 [51.9, 65.7]	56.5 [49.5, 63.4]	77.0 [71.1, 82.9]
	>2	25	24.7 [18.6, 30.7]	95.6 [92.7, 98.5]	80.0 [74.4, 85.6]	64.1 [57.4, 70.9]
	>3	5	6.2 [2.8, 9.6]	100.0 [100.0, 100.0]	100.0 [100.0, 100.0]	60.0 [53.1, 66.9]
	>4	0	n.a.	n.a.	n.a.	n.a.

The study was carried out in June 2010 in the Taabo health demographic surveillance system, south-central Côte d’Ivoire. Significance (p<0.05) of associations between risk factors, signs, and symptoms and parasites and treatment groups, respectively, was previously determined with multivariable logistic regression modeling, including a stepwise backward elimination. Participant’s combined score was obtained by coding all significantly associated risk factors, signs, and symptoms as 0 or 1 with the higher score indicating elevated odds for being infected with the respective helminth or belonging to a certain treatment group. The scores from all significantly associated risk factors, signs, and symptoms were then summed up to obtain each participant’s combined score.

a Tx1, treatment group 1 (i.e., praziquantel against schistosomiasis); Tx2, treatment group 2 (i.e., benzimidazole against soil-transmitted helminthiasis); Tx3, treatment group 3 (i.e., praziquantel and benzimidazole against schistosomiasis and soil-transmitted helminthiasis, respectively).

b CI, confidence interval.

n.a., not applicable as all individuals predicted as negative at the respective cut-off level.

## Discussion

In the present study, we juxtaposed quality-controlled parasitological data pertaining to schistosome and soil-transmitted helminth infections to sociodemographic data and detailed information on risk factors, signs, and symptoms. Data were obtained during a cross-sectional epidemiological survey conducted in mid-2010 in the Taabo HDSS in south-central Côte d’Ivoire. The intention of this study was twofold. First, we wanted to inform the local health authorities in the study area about risk factors, signs, and symptoms associated with schistosomiasis and soil-transmitted helminthiasis. Second, we were interested in the performance of an anamnestic questionnaire to predict helminth infection in an area subjected to preventive chemotherapy and a strengthened health system. Our study area might therefore represent a typical setting of low-endemicity helminthiasis due to sustained control interventions. Disappointingly, not a single risk factor, sign, or symptom, or combinations of them, revealed promising statistical associations and diagnostic properties in this ‘new parasitic landscape’ of control-induced low endemicity.

Nevertheless, some issues warrant further discussion. First, the sample size of our study was relatively small (n = 195 adults with complete parasitological and questionnaire data) and, with the exception of hookworm infection (39%), helminth prevalences were indeed very low (<5%). The heads of households and, if possible, a second adult household member of the opposite sex were purposefully sampled from over 1,100 participants who were enrolled during the cross-sectional epidemiological survey in the Taabo HDSS. Importantly, the prevalences of helminth infections in the cross-sectional epidemiological survey were similar to those in our study sample. In the overall survey, the prevalence of hookworm, *S. haematobium*, *S. mansoni*, *T. trichiura*, and *A. lumbricoides* were 31.3%, 3.7%, 2.1%, 1.6%, and 0.8%, respectively (E. K. N’Goran and colleagues, unpublished results).

Second, the low prevalence and intensity of helminth infection are the likely result of recent control efforts (i.e., annual deworming, health education, improved sanitation, and strengthened health system), which turned the previously polyparasitic study area into an area, where mainly hookworm infections remain. Indeed, N’Goran and colleagues reported a *S. haematobium* prevalence among school children in selected villages of 70% and above in the early and mid-1990s, and still in 2001 [32], [33], [43], whereas surveys conducted in 2008 and 2009 prior to Taabo HDSS-related deworming activities and other control interventions revealed hookworm prevalences of 51–89% [34], [35]. We can expect similar helminth prevalences in other communities where preventive chemotherapy campaigns are underway. The relative importance of hookworm *versus* the roundworm *A. lumbricoides* will likely be higher now as single-dose albendazole has a considerably lower efficacy in eliminating hookworm compared to *A. lumbricoides* infection [44]. For example, a recent study in Yunnan province, People’s Republic of China demonstrated a 67.1% prevalence of hookworm prior to single-dose albendazole, and a prevalence of 20.7% 3–4 weeks after treatment. In the same study, the prevalence of *A. lumbricoides* dropped from 95.1% to 3.7% [45]. With the scale up of preventive chemotherapy against helminthiases, it is likely that hookworm (and *T. trichiura*) infections will predominate in areas which were once highly endemic for schistosomiasis and soil-transmitted helminthiasis. Although our study area can now be considered a low endemicity region for schistosomiasis and a moderate endemicity area for soil-transmitted helminthiasis [29], it is a consequence of local helminth control efforts, and might therefore differ to other naturally occurring low or moderate endemic settings.

Third, as efforts are underway to integrate different control programs targeting multiple neglected tropical diseases [46], [47], questions arise as to how one might identify treatment-specific groups most efficiently. Hence, we did not only consider helminth-specific groups, but also treatment-specific groups. This is in line with recent WHO policies, which state that “preventive chemotherapy interventions should be conceived as drug-based rather than disease-based: emphasis should be on the best, coordinated use of the available drugs rather than on specific forms of helminthiasis” [18]. However, due to the low prevalence *Schistosoma* infection, our praziquantel treatment group (Tx1) also became comparatively small and due to the several fold higher prevalence of hookworm infection, our benzimidazole treatment group (Tx2), and our combined treatment group (Tx3) were clearly driven by the hookworm cases. Therefore, it is difficult to draw firm conclusions and further verification is needed to assess whether anamnestic questionnaires targeting at specific treatment groups may constitute a potential way forward in settings with higher levels of helminth co-endemicity. Also of note when further following a treatment group design, there are other intestinal parasites such as *Strongyloides stercoralis* [34], [35], [48], [49], which should be included in future considerations, and their rapid assessment and treatment within treatment groups of existing, efficacious, safe, often donated, or low-cost drug combinations [50], [51] may further enhance the usefulness of the approach.

Fourth, people reporting that worm infections are frequent in their household when directly asked showed higher odds of hookworm infection, and hence they were more likely to belong to two of the treatment groups considered here (Tx2 and Tx3). People may have some knowledge about helminth infections, possibly acquired during previous research and interventions in the region. If this hypothesis could be confirmed in future studies, the local control efforts should implicitly draw on this knowledge [52].

Fifth, we applied comparatively simple statistical models to analyze the data, including univariable and multivariable models and a scoring method, which is similar to approaches frequently applied in other health-related tests [53]–[56]. Interestingly, an approach using a scoring method and associated flexible score thresholds to predict positive cases may allow for the adaptation of the questionnaire’s sensitivity, specificity, and predictive values. However, better methods to elicit potentially useful anamnestic questions and their optimal combination would be needed.

Sixth, our anamnestic questionnaire did not perform well for predicting schistosomiasis and soil-transmitted helminth infections in the Taabo HDSS area where control interventions against helminthiases, other neglected tropical diseases, and malaria are underway. The lack of a clear and readily assessable epidemiological and clinical footprint in control-induced low-endemicity settings makes it difficult to inform local health workers and village authorities about the disease and to motivate them to keep up the disease control efforts. Such difficulties are inherent to control-induced low-endemicity settings and have also been noted on the “last mile” of dracunculiasis eradication [57]. Adapting a surveillance-response mechanism (i.e., surveillance for detection of new cases, followed by setting-specific health intervention in response to the cases picked up in the surveillance) should be considered as the way forward.

In conclusion, we recommend further studies in the Taabo HDSS, but also in other areas with currently still higher prevalences and intensities of helminth infections, to shed additional light on the scope and limits of anamnestic questionnaires as monitoring and guidance tool for neglected tropical disease control programs. Certainly, other clinical factors such as physical exam features and rapid diagnostic tests should be further evaluated as well. Likewise, improvements of alternative helminth diagnostics should be kept in mind in order to continuously appraise the optimal combination of the different diagnostic and monitoring tools. For instance, the Schistosomiasis Consortium for Operational Research and Evaluation (SCORE; http://score.uga.edu) conducted a multi-country study to assess the diagnostic accuracy of a commercially available point-of-care urine circulating cathodic antigen (POC-CCA) assay for the rapid detection of *S. mansoni* infection, which showed that POC-CCA urine tests are more sensitive than routine Kato-Katz thick smears [58]. Combining features of the different diagnostic and monitoring tools in consideration of the setting-specific levels of endemicity of the different parasites will become more important as prevalence rates and infection intensities decline due to successful morbidity and infection control and such combinations will likely result in the most efficient care at the point-of-contact. Of note, the European research network NIDIAG is currently aiming at the development of improved, simple, integrated, and cost-effective diagnosis and treatment algorithms for clinical syndromes related to neglected tropical diseases (NIDIAG; http://www.nidiag.org) and such more comprehensive approaches may also provide the necessary framework for the additional evaluation and potential application of anamnestic questionnaires.

## Supporting Information

Text S1 **Questionnaire for evaluating the health state of individuals in June 2010 in the Taabo health demographic surveillance system, south-central Côte d’Ivoire (in French).**
(DOC)
